# Editorial: Microbial and Environmental Factors in Autoimmune and Inflammatory Diseases

**DOI:** 10.3389/fimmu.2017.00243

**Published:** 2017-03-06

**Authors:** Marina I. Arleevskaya, Gayane Manukyan, Ryo Inoue, Rustam Aminov

**Affiliations:** ^1^Central Research Laboratory, Kazan State Medical Academy, Kazan, Russia; ^2^Group of Molecular and Cellular Immunology, Institute of Molecular Biology, National Academy of Sciences, Yerevan, Armenia; ^3^Laboratory of Animal Science, Department of Agricultural and Life Sciences, Kyoto Prefectural University, Kyoto, Japan; ^4^School of Medicine and Dentistry, University of Aberdeen, Aberdeen, UK

**Keywords:** rheumatoid arthritis, autoimmune ecology, silica exposure, viruses, bacteria, environment

**The Editorial on the Research Topic**

**Microbial and Environmental Factors in Autoimmune and Inflammatory Diseases**

Pathogenesis of many autoinflammatory and autoimmune diseases is largely driven by inappropriate or inadequate immune responses toward environmental challenges. Most of them are multifactorial. For example, assessment of disease risk factors in rheumatoid arthritis (RA) brings the share of genetic predisposition to 50–60%, with the rest attributed to environmental factors (1). Thus, the diseases such as RA are well defined by Meda et al. (2) as comprising of both genetic and epigenetic components: “the disease of bad genes and bad luck.” While genetic predisposition to RA and other autoimmune and autoinflammatory diseases has been a subject of very extensive research in recent years, the role of environmental factors remains less explored. The research topic articles address the role of various environmental factors that may contribute to the onset of autoinflammatory and autoimmune disorders. They are presented in the following order: purely environmental factors, dysbiotic conditions, and infections of bacterial, fungal, and viral nature.

In their comprehensive review, Anaya et al. discuss what they call the “autoimmune ecology,” which includes all levels involved, from the ecosphere to the environmental factors and to the molecular mechanisms of interaction of these factors with the human immune system. The role of microbiota and vaccines, lifestyle habits and socioeconomic status, organic solvents, ultraviolet, and other factors is discussed in the context of how these factors may interact with the innate immunity *via* the toll-like receptor signaling pathway, B-cell activation, the T helper 17, and regulatory T (Treg) cells, posttranslational modifications of self-antigens, and epigenetic modifications. This is a formidable attempt to synthetize the current knowledge of how various environmental factors (collectively called exposome) may interact with the immune system leading to differential outcomes including autoimmune disease.

In the perspective article by Pollard, the silica-induced trigger mechanisms in the pathogenesis of inflammatory and autoimmune diseases are overviewed. Despite the proven epidemiological link between the silica exposure and autoimmune diseases, very little is known about the mechanistic factors leading to this disease. Based on currently available human and animal studies, a putative chain of events is proposed, which begins with activation of the innate immune system and then leads to proinflammatory cytokine production, pulmonary inflammation, activation of adaptive immunity, breaking of tolerance, autoantibody production, and tissue damage. Animal models of the silica exposure, which are used to mimic human autoimmunity, are also discussed.

In systemic lupus erythematosus (SLE), with the disease onset in adulthood, the preceding prenatal/maternal history could bear important risk factors (Parks et al.). The authors demonstrate specific links between SLE and some early-life factors such as preterm birth, low birth weight, early prenatal/maternal farm exposure, and extended farm residence in childhood.

Several articles highlight a potential link between autoimmune diseases and the gastrointestinal tract disorders such as dysbiosis and bacterial infections. A review article by Ignacio et al. overviews host–microbe interaction and how the innate immune system senses gut microbiota and their metabolites *via* the inflammasomes and toll-like receptors. Disturbances in this fine-tuned cross talk may cause inappropriate modulations of inflammatory pathways thus leading to local and systemic inflammatory and autoimmune diseases.

In their hypothesis and theory article Lerner et al. propose an interesting mechanism, which may explain why a dysbiotic microbiota may have a potential role in autoimmune diseases. Physiologically normal posttranslational modifications of proteins by gut microbiota include cross-linking, de/amination/deamidation, de/phosphorylation, a/deacetylation, de/tyrosination, de/glutamylation, de/glycylation, ubiquitination, palmitoylation, glycosylation, galactosylation, arginylation, methylation, citrullination, sumoylation, carbamylation, and others. The balance of these activities in dysbiosis may be compromised thus generating highly immunogenic or neo-epitopes, which may break the tolerance and induce autoimmunity.

In the opinion article by Saxena, probiotic treatment is proposed as an alternative to the conventional treatment of Guillain–Barré syndrome, an immune-mediated peripheral neuropathy. The main idea is that probiotics may help to normalize the dysbiotic microbiota and replenish Treg cells to promote immune homeostasis.

Regarding gastrointestinal infections, Khaiboullina et al. demonstrate that gastroduodenitis caused by *Helicobacter pylori* is due to the upregulation of serum chemokines CXCL5 and CXCL6. This may lead to the local neutrophil accumulation at the sites of inflammation. *H. pylori* can also be protective against allergy and asthma, and Hussain et al. demonstrate a potential link between the human systemic type 1 T helper and Treg responses to *H. pylori* and allergen-specific IgE levels. The conclusion is that the systemic IL-10^+^ Treg response is likely to play a role in *H. pylori*-mediated protection against allergy in humans.

Chronic infections of the upper gastrointestinal tract can also provoke autoimmune conditions. Fuggle et al. performed extensive meta-analysis of data extracted from relevant publications to elucidate a potential link between RA and periodontitis. There is indeed a significant association between these two conditions. However, when we compare RA and osteoarthritis as a control, there is no significant difference in the prevalence of periodontitis in these two groups.

Some viral infections are the potent inducers of immune response, while others such as hantavirus infection are considered as having a much lesser impact. However, Morzunov et al. demonstrate a differential expression of serum cytokines involved in migration of lymphocytes, natural killer cells, and dendritic cells to the sites of hantavirus infection. There is also a significant upregulation of cytokines regulating leukocyte migration, repair of lung tissue, permeability of endothelial monolayer, and transendothelial leukocyte migration. On the contrary, cytokines associated with the platelet numbers and functions are downregulated. The same research group also reviews the hantaviral proteins and their potential involvement in hantavirus infection (Muyangwa et al.).

Another article from the same group (Boichuk et al.) shows the loss of plasmacytoid dendritic cells (pDCs), which are the major producers of interferon (IFN) type I, in the periphery of subjects with HIV/AIDS. This is presumably due to the decrease in serum cytokines. At the same time, gut-associated pDCs in HIV express the cellular proliferation marker Ki-67 suggesting that gut-associated pDCs are naive and possibly of the bone marrow origin. Gut-associated pDCs display activated phenotypes, with the elevated level of the proapoptotic enzyme, granzyme B. These data support a model of HIV progression where the peripheral pDCs are depleted and replaced by naive bone marrow-derived pDCs with limited IFN-producing capabilities. Ultimately, these pDCs migrate to the gut, with a potential impact on the gut mucosa due to the production of inflammatory cytokines and granzyme B.

Attempts to develop novel treatments against viral diseases such as swine flu, which is caused by the subtype of influenza A virus (H1N1), include the exploration of potentials of traditional medicine (Romero-Pérez et al.). The authors demonstrate some positive effects of stems and roots of *Salacia reticulate* (used in traditional Indian medicine Ayurveda) in a mouse model of H1N1 infection. These effects are presumably due to the increased natural killer cell activity in the host.

A potential role of common microbial and viral infections in the onset of RA is reviewed by Arleevskaya et al. The authors conclude that the optimal immune response toward these infections could be achieved *via* its reasonable adequacy and fine balance of immune system components. In some patients, however, this delicate equilibrium is compromised thus leading to the onset of RA. Contributing factors among these patients could be (i) higher susceptibility to bacterial and viral infections; (ii) greater imbalance of immune system components; (iii) limited capability to control and resolve inflammation; and (iv) compromised interaction at the microorganism–immune system interface. Thus, the disease onset is driven by the combination of genetic and environmental factors.

An important event in launching an appropriate immune response is sensing the invading pathogens by the innate immune system, and one of the important components of it is a protein called pyrin. Manukyan and Aminov give an up-to-date review of structure and function of this protein. They also examined molecular mechanisms of pathogenesis of familial Mediterranean fever, one of the most common hereditary autoinflammatory syndromes caused by mutations in pyrin. The high carrier frequency found in the affected populations from the Mediterranean Basin suggests a selective advantage conferred by the heterozygous state such as protection against presumptive pathogen(s). Identity of the suspected pathogen(s) that had selected for this genetic trait, however, remains elusive.

Another sensing molecule brought up in this topic is pentraxin-3, which serves as a key soluble pattern recognition receptor as well as interacts with the components of complement pathway. Hence, it plays an important role in innate immune responses against several bacterial, fungal, and viral infections. Computational analysis of non-synonymous single nucleotide polymorphisms (nsSNPs) in the *PTX-3* gene suggests that there are 10 high-risk nsSNPs (Thakur and Shankar). Four of them affect the conserved structural and functional residues in the pentraxin-domain thus compromising interaction with the C1q component of complement pathway. This may result in an increased susceptibility to infections among the carriers of these nsSNPs.

SNP-associated risk factors are not necessarily limited to structural/regulatory proteins involved in pathogen sensing, signal transduction, or immune response. An *in silico* analysis of 72 SNPs associated with 43 primary Sjögren’s syndrome (SS) genetic risk factors suggests that only 5.6% are associated with the coding sequences (Konsta et al.). Other SNPs include intron sequences (55.6%), regions upstream/downstream of genes (30.5%), and intergenic regions (8.3%). Consequently, a significant enrichment by regulatory motifs (promoter, enhancer, insulator, DNAse peak, and expression quantitative trait loci) characterizes risk variants for SS (94.4%). Analysis of risk variants for SS among histone markers in B cells, monocytes, and epithelial cells shows their close association with promoters and enhancers in B cells and with enhancers in monocytes.

In conclusion, articles in this research topic made a very significant contribution to our understanding of the role played by environmental factors, dysbiotic conditions, and infections in triggering autoimmune and autoinflammatory diseases. Since this is a rapidly expanding area of research, many other factors contributing to the onset of these diseases are not covered here. We are confident, however, that further studies will expand the list as well as bring a better understanding of mechanisms involved in the onset of autoimmune and autoinflammatory diseases.

## Author Contributions

All authors listed have made substantial, direct, and intellectual contribution to the work and approved it for publication.

## Conflict of Interest Statement

The authors declare that the research was conducted in the absence of any commercial or financial relationships that could be construed as a potential conflict of interest.
